# Sex differences in the 10-year survival of patients undergoing maintenance hemodialysis in the Q-Cohort Study

**DOI:** 10.1038/s41598-021-03551-x

**Published:** 2022-01-10

**Authors:** Hiroaki Tsujikawa, Shunsuke Yamada, Hiroto Hiyamuta, Masatomo Taniguchi, Kazuhiko Tsuruya, Kumiko Torisu, Toshiaki Nakano, Takanari Kitazono

**Affiliations:** 1 Department of Medicine and Clinical Science, Graduate School of Medical Sciences, Kyushu University, 3-1-1 Maidashi, Higashi-ku, Fukuoka, 812-8582 Japan; 2 Fukuoka Renal Clinic, Fukuoka, Japan; 3 Department of Nephrology, Nara Medical University, Nara, Japan; 4 Department of Integrated Therapy for Chronic Kidney Disease, Graduate School of Medical Sciences, Kyushu University, Fukuoka, Japan

**Keywords:** Nephrology, Prognosis

## Abstract

Women have a longer life expectancy than men in the general population. However, it has remained unclear whether this advantage is maintained in patients undergoing maintenance hemodialysis. The aim of this study was to compare the risk of mortality, especially infection-related mortality, between male and female hemodialysis patients. A total of 3065 Japanese hemodialysis patients aged ≥ 18 years old were followed up for 10 years. The primary outcomes were all-cause and infection-related mortality. The associations between sex and these outcomes were examined using Cox proportional hazards models. During the median follow-up of 8.8 years, 1498 patients died of any cause, 387 of whom died of infection. Compared with men, the multivariable-adjusted hazard ratios (95% confidence interval) for all-cause and infection-related mortality in women were 0.51 (0.45–0.58, *P* < 0.05) and 0.36 (0.27–0.47, *P* < 0.05), respectively. These findings remained significant even when propensity score-matching or inverse probability of treatment weighting adjustment methods were employed. Furthermore, even when the non-infection-related mortality was considered a competing risk, the infection-related mortality rate in women was still significantly lower than that in men. Regarding all-cause and infection-related deaths, women have a survival advantage compared with men among Japanese patients undergoing maintenance hemodialysis.

## Introduction

Women have a longer life expectancy than men in the general population^1^. The World Health Organization’s analysis of global health statistics according to sex clearly show that women have better longevity prospects than men^2^. This difference between men and women is related to various factors including genetic and physiological ones, such as the progressive skewing of X-chromosome inactivation^3^, telomere attrition^4^, mitochondrial inheritance^5^, hormonal and cellular responses to stress^6^, and immune function^7,8^.

Regarding patients undergoing maintenance hemodialysis (HD), conflicting data have been reported on the survival advantage of women over men. Some studies reported that men tend to be more susceptible than women to uremia and inflammation-induced anorexia^9^. Furthermore, inflammatory and nutritional variables may deteriorate over time in men^10^. Women with inflammation undergoing HD were reported to have lower mortality rates than men with the same status^11^. Conversely, other studies have indicated similar mortality rates between women and men undergoing HD^12–14^_._ Additionally, several studies showed that infection-related mortality was higher in women undergoing HD than in men undergoing HD^15–17^. By analyzing the data from the international Dialysis Outcomes and Practice Patterns Study (DOPPS), Hecking et al*.* observed higher catheter use among women and a significant sex-specific association of catheter use with mortality, specifically, a higher risk of mortality for women having a catheter as hemodialysis access than for men^15^. In the Japanese DOPPS, the prevalence of catheter use was much lower than that in other countries. These results might indicate that the general survival advantage for women over men may be nullified by the high prevalence of HD catheter use and resulting mortality in women due to infection. Considering that the prevalence of HD catheter use in Japan is lower than that in other countries, it would be reasonable to examine the female survival advantage in Japanese HD patients by focusing on infection-related mortality^18^.

Against this background, the current study investigated whether there is a difference between the sexes in the risk of mortality, especially infection-related mortality, among HD patients. In pursuit of this aim, we analyzed the dataset of the Q-Cohort Study, a multicenter, observational cohort study of Japanese patients undergoing maintenance HD^19^, by using conventional Cox proportional hazards models and propensity score (PS)-based statistical analysis.

## Results

### Baseline characteristics of the patients stratified by sex

The baseline characteristics of the patients stratified by sex are shown in Table 1. Women were significantly (*P* < 0.05) older and had a longer dialysis vintage, lower frequency of diabetic nephropathy, and lower frequency of cardiovascular disease (CVD) history. The cardiothoracic ratio, normalized protein catabolic rate (nPCR), single-pool Kt/V for urea, and serum concentrations of total cholesterol, albumin-corrected calcium (Ca), and alkaline phosphatase were significantly (*P* < 0.05) higher in women than those in men. Conversely, the body weight, blood hemoglobin level, serum concentrations of urea nitrogen, creatinine, and albumin, and frequencies of antihypertensive agent and vitamin D receptor activators (VDRA) use were significantly lower in women than those in men.

**Table 1:** Baseline characteristics according to sex.

Variables	Men (n = 1816)	Women (n = 1249)	*P* value
Age (years)	63.9 (55.8–71.7)	64.7 (55.9–74.0)	0.011
Diabetic nephropathy, n (%)	593 (32.7)	296 (23.7)	< 0.001
History of CVD, n (%)	619 (34.1)	342 (27.4)	< 0.001
Dialysis vintage (years)	5.0 (2.0–10.5)	5.4 (2.1–12.3)	0.020
Dialysis time (hours)	5 (4–5)	5 (4.5–5)	0.005
Body weight (kg)	57.5 (51.4–64.3)	46.4 (41.0–52.5)	< 0.001
Systolic blood pressure (mmHg)	156 (142–169)	150 (134–168)	< 0.001
Cardiothoracic ratio (%)	49.1 (46.0–52.4)	51.9 (48.1–55.2)	< 0.001
nPCR (g/kg/day)	0.94 (0.82–1.03)	0.97 (0.87–1.10)	< 0.001
Single-pool Kt/V for urea	1.48 (1.32–1.58)	1.74 (1.56–1.96)	< 0.001
Blood hemoglobin (g/dL)	10.6 (9.9–11.4)	10.4 (9.7–11.1)	< 0.001
Serum urea nitrogen (mg/dL)	66 (56–76)	67 (57–77)	0.278
Serum creatinine (mg/dL)	11.1 (9.1–12.9)	9.2 (7.7–10.7)	< 0.001
Serum total cholesterol (mg/dL)	144 (125–166)	166 (145–191)	< 0.001
Serum albumin (g/dL)	3.8 (3.6–4.1)	3.8 (3.6–4.0)	0.006
Serum CRP (mg/dL)	0.13 (0.07–0.32)	0.12 (0.04–0.27)	< 0.001
Albumin-corrected serum Ca (mg/dL)	9.3 (8.8–9.9)	9.5 (9.0–10.0)	< 0.001
Serum phosphate (mg/dL)	4.9 (4.2–5.6)	4.9 (4.2–5.7)	0.693
Serum alkaline phosphatase (U/L)	224 (174–292)	253 (194–343)	< 0.001
Serum PTH (pg/mL)	107 (50–207)	103 (45–220)	0.808
Use of antihypertensive agents, n (%)	1220 (67.2)	717 (57.4)	< 0.001
Dose of ESAs, unit/week	3000 (1500–4500)	3000 (1500–4500)	0.007
Use of phosphate binders, n (%)	1512 (83.3)	1017 (81.4)	0.192
Use of VDRAs, n (%)	1338 (73.7)	838 (67.1)	< 0.001

Values are presented as median (interquartile range) for continuous variables and number (percentage) for categorical variables.

*Ca* calcium, *CRP* C-reactive protein, *CVD* cardiovascular disease, *ESA* erythropoiesis-stimulating agent, *nPCR* normalized protein catabolic rate, *PTH* parathyroid hormone, *VDRA* vitamin D receptor activator.

### The risk of all-cause and infection-related mortality stratified by sex

**Figure 1:**
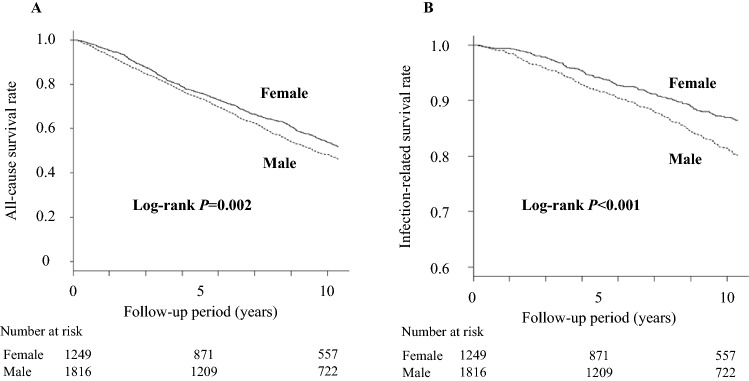
Kaplan–Meier curves for all-cause (**A**) and infection-related (**B**) mortality stratified by sex. The 10-year incidence rates of all-cause mortality and infection-related mortality in women were significantly lower than those in men (*P* < 0.05). A log-rank test was used to determine whether there was a significant difference between women and men. A *P*-value of less than 0.05 was considered statistically significant.

During the 10-year follow-up, 1498 patients (48.9%) died of any cause, among which 387 patients (12.6%) died of an infection-related cause, 542 patients (17.7%) died of cardiovascular disease, and 204 patients (6.7%) died of malignancy. The unadjusted 10-year incidence rate of all-cause mortality was significantly lower in women than in men (*P* < 0.001) (Fig. 1A). A previous observational study reported that the crude all-cause mortality rate was 9.06 per 1000 person-years in the Japanese general population and 94.10 per 1000 person-years in the Japanese dialysis population for 2008 and 2009^20^. In our study, the crude all-cause mortality rate was 68.9 per 1000 person-years, roughly consistent with the rate reported earlier. Furthermore, women had a lower risk of all-cause death than men after adjustment for all variables: the hazard ratio (HR) (95% confidence interval (CI)) was 0.51 (0.45–0.58, *P* < 0.001; Table 2).

**Table 2:** Hazard ratios and 95% CIs for all-cause mortality.

	Hazard ratio (95% CI) (versus men)	*P-value*
Age-adjusted Cox model	0.74 (0.66–0.82)	< 0.001
Fully adjusted Cox model	0.51 (0.45–0.58)	< 0.001
PS-matching model (1 : 1, n = 1322)	0.49 (0.42–0.58)	< 0.001
IPTW model (n = 3065)	0.61 (0.53–0.69)	< 0.001

The fully adjusted model included the following covariates: age, presence of diabetic nephropathy, history of CVD, dialysis vintage, pre-dialysis systolic blood pressure, body weight, cardiothoracic ratio, nPCR, single-pool Kt/V for urea, blood hemoglobin, serum concentrations of urea, creatinine, total cholesterol, albumin, CRP, albumin-corrected serum Ca, phosphate, alkaline phosphatase, and PTH, dose of ESAs, and use of antihypertensive drugs, phosphate binders, and VDRAs. The PS-matching model was adjusted for body weight and serum creatinine concentration. The IPTW model weighted patients by PS and adjusted for body weight and serum creatinine concentration.

*Ca* calcium, *CI* confidence interval, *CRP* C-reactive protein, *CVD* cardiovascular disease, *ESA* erythropoiesis-stimulating agent, *IPTW* inverse probability of treatment weighting, *nPCR* normalized protein catabolic rate, *PS* propensity score, *PTH* parathyroid hormone, *VDRA* vitamin D receptor activator.

Next, we determined the association between sex and infection-related death. The unadjusted 10-year incidence rate of infection-related death in women was significantly decreased compared with that in men (P < 0.001) (Fig. 1B). Women had a lower risk of infection-related death than men after adjustment for all variables: the HR (95% CI) was 0.36 (0.27–0.47, *P* < 0.001; Table 3). Furthermore, even when the competing event of non-infection-related death was considered, the infection-related mortality rate in women was significantly lower than that in men: the HR (95% CI) was 0.46 (0.35–0.60, *P* < 0.001).

**Table 3:** Hazard ratios and 95% CIs for infection-related mortality.

	Hazard ratio (95% CI) (versus men)	*P-value*
Age-adjusted model	0.57 (0.49–0.70)	< 0.001
Fully adjusted model	0.36 (0.27–0.47)	< 0.001
Fine & Gray model	0.46 (0.35–0.60)	< 0.001
PS-matching model (1 : 1, n = 1416)	0.31 (0.23–0.43)	< 0.001
IPTW model (n = 3065)	0.51 (0.44–0.58)	< 0.001

The fully adjusted model included the following covariates: age, presence of diabetic nephropathy, history of CVD, dialysis vintage, pre-dialysis systolic blood pressure, body weight, nPCR, single-pool Kt/V for urea, blood hemoglobin, serum concentrations of urea, creatinine, total cholesterol, albumin, CRP, albumin-corrected serum Ca, phosphate, alkaline phosphatase, and PTH, dose of ESAs, and use of phosphate binders and VDRAs. The Fine & Gray model with non-infection-related deaths as a competing risk was used to consider the competing risk. The PS-matching model was adjusted for body weight and serum creatinine concentration. The IPTW model weighted patients by PS and adjusted for body weight and serum creatinine concentration.

*Ca* calcium, *CI* confidence interval, *CRP* C-reactive protein, *CVD* cardiovascular disease, *ESA* erythropoiesis-stimulating agent, *IPTW* inverse probability of treatment weighting, *nPCR* normalized protein catabolic rate, *PS* propensity score, *PTH* parathyroid hormone, *VDRA* vitamin D receptor activator.

Regarding cardiovascular mortality, there was no significant difference in the 10-year incidence rate between women and men (Supplementary Fig. 1A). Moreover, regarding malignancy-related death, the 10-year incidence rate in women was significantly lower than that in men (*P* = 0.002) (Supplementary Fig. 1B).

### The risk of all-cause and infection-related mortality analyzed by the PS-matching method and inverse probability of treatment weighting (IPTW) adjustment method

To further focus on the female survival advantage in Japanese HD patients regarding all-cause and infection-related mortality, we conducted additional analyses by applying PS-based approaches to all-cause and infection-related mortality. The logistic regression model used in the PS analysis for all-cause and infection-related mortality showed high discriminatory power with area under the receiver operating characteristic curve values of 0.86 and 0.84, respectively. The imbalances of baseline covariates in the pre-matching cohort were well resolved after adjustment with the PS-matching method (Supplementary data, Tables S1 and S2). Serum creatinine concentration and body weight were not included in the creation of the PS; however, these two covariates are regarded as inherent characteristics of sex differences and were thus balanced across the sexes after using the PS methodology. Notably, the survival advantage of women remained statistically significant even when the PS-matching and IPTW methods were employed (Tables 2 and 3).

### Subgroup IPTW analyses stratified by baseline clinical characteristics

**Figure 2:**
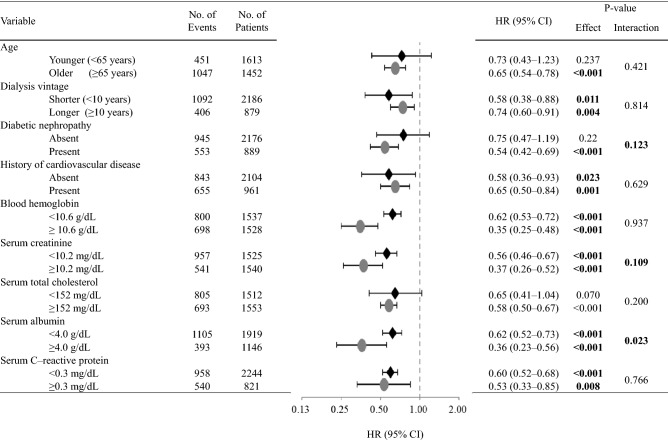
The propensity-weighted HRs and 95% CIs for all-cause mortality in women compared with men according to the subgroups of baseline characteristics adjusted using Cox regression analysis in propensity score-weighted samples. Adjustment was performed for baseline characteristics (age, presence of diabetic nephropathy, history of cardiovascular disease, dialysis vintage, blood hemoglobin level, and serum concentrations of creatinine, total cholesterol, albumin, and C-reactive protein). A *P*-value of less than 0.05 was considered statistically significant. *CI* confidence interval, *HR* hazard ratio.

**Figure 3:**
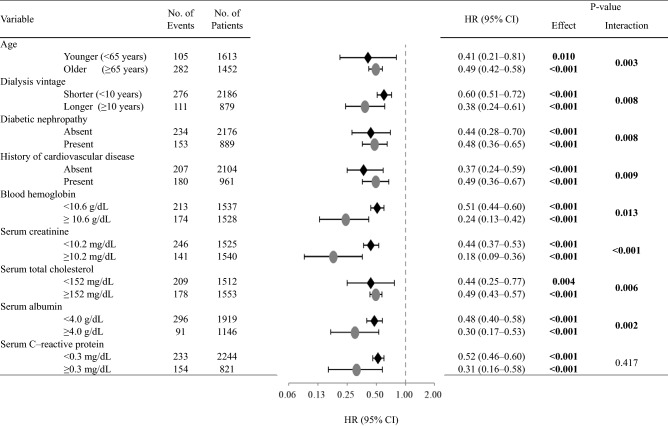
Propensity-weighted HRs and 95% CIs for infection-related mortality in women compared with men according to subgroups of baseline characteristics adjusted using Cox regression analysis in propensity score-weighted samples. Adjustment was performed for baseline characteristics (age, presence of diabetic nephropathy, history of cardiovascular disease, dialysis vintage, blood hemoglobin level, and serum concentrations of creatinine, total cholesterol, albumin, and C-reactive protein). A *P*-value less than 0.05 was considered statistically significant. *CI* confidence interval, *HR* hazard ratio.

To assess whether the survival benefit for women is consistent across a variety of baseline clinical backgrounds, the effects upon classification into subgroups stratified by potential confounders at baseline were examined using the IPTW method (Figs. 2 and 3). The finding of a lower rate of all-cause death in women was more robust in patients with presence of diabetic nephropathy or higher serum creatinine or albumin concentration (Fig. 2). In addition, in women, the risk of infection-related death tended to be lower in younger patients, patients with longer dialysis vintage, or patients with non-diabetic nephropathy, no history of CVD, or higher levels of blood hemoglobin, higher serum levels of creatinine or albumin, or lower levels of serum total cholesterol (Fig. 3).

## Discussion

In the present study, by employing various statistical approaches, we clearly demonstrated a survival advantage of women over men for both all-cause and infection-related deaths in patients undergoing HD. Subgroup analysis also revealed that women’s risk of all-cause mortality was lower than that of men among patients with diabetic nephropathy or higher serum levels of creatinine or albumin. Moreover, women’s infection-related mortality risk was lower than that of men who were younger, with longer dialysis vintage, with non-diabetic nephropathy, no history of CVD, higher levels of blood hemoglobin, higher serum levels of creatinine or albumin, or lower levels of serum total cholesterol*.* Taken together, our results suggest a potential survival benefit for female patients undergoing maintenance HD.

The present study has provided evidence that hemodialyzed women have a survival advantage over hemodialyzed men. To the best of our knowledge, our study is the first to demonstrate that the female survival advantage is also present specifically for infection-related mortality in HD patients. This relationship remained statistically significant even after adjustment for potential confounding factors, PS-matching, or IPTW adjustment. Furthermore, when non-infection-related death was considered a competing risk, the infection-related mortality rate in women was significantly lower than that in men. As for all-cause death, a report from the DOPPS demonstrated that the HR (95% CI) of all-cause mortality in men (versus women) was 1.09 (1.04–1.14) after adjusting for age and time on dialysis^13^, consistent with our current observations. Taken together, our data and previous reports suggest that women have a survival advantage over men when they are on maintenance HD.

Several potential mechanisms might explain this survival advantage of women over men undergoing HD. Previous studies reported that, in comparison to female patients undergoing HD, men might be more susceptible to inflammation-induced anorexia and can exhibit more severe symptoms (e.g., handgrip strength decline^9^) and deterioration over time, as evidenced by nutritional and inflammatory variables such as albumin, body weight, C-reactive protein (CRP), and interleukin-6^10^. It has also been demonstrated in regard to inflammation that women have better outcomes than men^11^. These results suggest that men are more vulnerable than women in the HD population. In the general population, mounting evidence has also shown that the survival advantage of women is related to genetic and physiological factors. Specifically, inactivation of the disadvantageous X chromosome^3^, longer telomeres^4^, a lower resting metabolic rate^21^, estrogen^22^, and mitogenome–nuclear genome interactions^6^ might play a role in the greater longevity of women. These factors could partly explain the mechanism underlying our observations. Furthermore, the heightened immune response in women is generally considered to make them more resistant to infections^7,8,23^. Our study confirmed a similar relationship in patients undergoing maintenance HD.

The subgroup analysis of all-cause mortality revealed that women’s all-cause mortality risk was significantly lower than that of men among those with diabetic nephropathy or higher serum levels of creatinine or albumin. Additionally, the subgroup analysis of infection-related mortality revealed that women’s infection-related mortality risk was lower than that of men who were younger, with longer dialysis vintage, with non-diabetic nephropathy, no history of CVD, higher levels of blood hemoglobin, higher serum levels of creatinine or albumin, or lower levels of serum total cholesterol. In our analysis, women’s mortality risk in diabetic nephropathy was different in each outcome. Previous studies have shown that the age-related declines of immune cells and inflammatory mediators were slower in women than in men^7,8^. Furthermore, women’s sex hormones might reduce antioxidants^22^, and women are more resistant to anorexia and malnutrition^9,10^. However, recent observational studies demonstrated the opposite association between sex and death rate in younger patients undergoing HD^17,24^. Hence, further studies are necessary to elucidate whether the effects of the baseline factors observed in the current study are present across a variety of HD populations and whether their underlying mechanisms are related to sex hormones.

Despite the accumulation of findings, women’s advantage regarding the life expectancy of patients undergoing HD is still controversial. Sex-dependent differences in the proportion of types of vascular access might partially explain the discrepant results. The results from the DOPPS revealed that the selection of vascular access differed between the sexes, with catheter use being less frequent in male HD patients (12.2%) than in female ones (18.4%); subgroup analyses indicated that HD catheter use was associated with a higher risk of all-cause mortality in female patients undergoing HD^13^. As catheter users are likely to develop catheter-related infections and resulting persistent inflammation followed by malnutrition, it is possible that they are at increased risk of infection-related or all-cause death. In this regard, sex differences in the proportion of types of vascular access may be important confounders that might have nullified the natural specific advantage of women. Importantly, a national survey conducted in 2008 in Japan reported that more than 90% of patients undergoing maintenance HD used an arteriovenous fistula, while only 0.5% used a catheter^18^. Additionally, there was no difference between the sexes in the proportion of types of vascular access in Japanese patients in the DOPPS^13^. This suggests that catheter users were a minor component in our study and that there is no sex discrepancy in the proportion of types of vascular access. In the present study, even when PS-matching or IPTW adjustment were employed, the survival advantage of women was statistically significant, particularly regarding all-cause and infection-related mortality. This indicates that catheter use during HD might diminish the natural specific advantage of women. However, it is impossible to assess this in the present study because we had no direct data regarding the type of vascular access. There is, therefore, a need for further studies, including data on the type of vascular access, to determine whether women undergoing HD have a survival advantage regardless of the type of vascular access.

The main strengths of our study were its large scale and wide-ranging inclusion criteria. As such, our results are generalizable to real-world HD patients. However, some limitations of our study should be noted. First, the measurements of baseline parameters might have been insufficient. For instance, data regarding the use of steroid or immunosuppressive agents and the acceptance rate of renal replacement therapy were missing. Second, we had no data on the serum levels of sex hormones. A previous study showed that women undergoing HD had lower serum estradiol levels than those in the general population^25^. Thus, the activity of sex hormones at one point in time when undergoing HD might hardly explain the discrepancy in mortality. The length of exposure to female hormones before HD initiation may determine the impact of the female advantage on survival. Third, the patients participating in this study were all Japanese; our results might not be applicable to other ethnic groups. Fourth, data regarding the infection rates in both sexes were not available in the present study. Hence, there was no evidence for whether women suffer fewer infections than men or whether the mortality rate after infection differed between men and women. Further studies are needed to confirm whether the observed difference in the infection-related mortality rate between the sexes was caused by the difference in the infection rate or the mortality rate after infection. Despite these limitations, we believe that this study provides further evidence that women have a survival advantage over men undergoing maintenance HD.

In conclusion, our findings on patients undergoing maintenance HD suggest that women’s risk of all-cause and infection-related death is lower than that of men. Further studies are required to confirm this female survival advantage and its underlying mechanisms under maintenance HD.

## Methods

### Study design and population

The details regarding the design of the Q-Cohort Study were described previously^18^. We recruited 3598 outpatients aged 18 years or older who were receiving maintenance HD in 39 HD facilities between 31 December, 2006, and 31 December, 2007. Participants were followed up until 31 December 2016. The participants’ health status was checked annually by local physicians at each dialysis facility. When patients moved to other HD facilities in which collaborators of this study were not present, we conducted follow-up surveys by mail or telephone. We excluded 533 participants with missing data on one or more baseline characteristics and whose outcome information could not be obtained. We enrolled the remaining 3065 patients in the final study population. The study protocol was approved by the Clinical Research Ethics Committee of the Institutional Review Board of Kyushu University (Approval Number 20-31) and all participating institutions. First, we followed the patients from 2006 until 2010. Written informed consent was obtained from all participants at the start of the study. The present study was performed in accordance with the Declaration of Helsinki. We subsequently surveyed the patients’ further follow-up from 2011 to 2016. The ethics committees of all participating institutions waived the requirement for written informed consent for the additional follow-up surveys from 2011 to 2016 because of the retrospective nature of the study. The study is registered in the University Hospital Medical Information Network (UMIN) clinical trial registry (UMIN ID: 000000556).

### Covariates

The main factor assessed was sex, and the potential confounders were as follows: age, presence of diabetic nephropathy, history of CVD, dialysis vintage, pre-dialysis systolic blood pressure, body weight, cardiothoracic ratio, nPCR, single-pool Kt/V for urea, blood hemoglobin level, serum concentrations of urea nitrogen, creatinine, total cholesterol, albumin, CRP, Ca, phosphate, alkaline phosphatase, and parathyroid hormone (PTH), dose of erythropoiesis-stimulating agents (ESAs), and use of antihypertensive agents, phosphate binders, and VDRAs. The ESA dosage for darbepoetin alfa administration was calculated by multiplying the dosage (µg) of darbepoetin alfa by 200. The above data were collected by reviewing medical records. Blood samples were collected before starting each HD session. The serum albumin-corrected serum Ca concentration was calculated using Payne’s formula: corrected Ca (mg/dL) = observed total Ca (mg/dL) + (4.0 – serum albumin concentration [g/dL]), only if the serum albumin concentration was < 4.0 g/dL. Serum PTH levels were measured using whole or intact PTH assays and expressed as intact PTH assay levels^26^.

### Definition of outcomes

The primary outcomes were all-cause and infection-related death as recorded in the patients’ medical records.

### Statistical analysis

Group differences in continuous variables were determined using the Mann–Whitney U test; categorical variables were compared using the chi-squared test. The incidence rates and 95% CIs for all-cause and infection-related mortality were calculated using the person-year method. The unadjusted, age-adjusted, and fully adjusted HRs with 95% CIs of all-cause and infection-related mortality according to sex were calculated using a Cox proportional hazards model. The fully adjusted model for all-cause mortality was adjusted for the abovementioned potential confounders. The fully adjusted model for infection-related mortality was adjusted for the same factors except the cardiothoracic ratio and use of antihypertensive agents. To adjust the selection bias by sex, we used the PS methodology^27^. The PS was calculated for each patient using a multivariable-adjusted logistic regression model with sex as the dependent variable. To analyze all-cause and infection-related mortality and calculate the PS, the same covariates as the abovementioned potential confounders were selected. The discriminatory power of the PS was evaluated by calculating the area under the receiver operating characteristic curve. A PS-matching model with adjustment for body weight and serum creatinine was employed to compare the impact of sex on mortality independently of potential confounders. The IPTW model was applied to weight patients by the PS and was adjusted for body weight and serum creatinine concentration. Statistical analyses were performed using R version 3.6.1 (http://www.r-project.org). A two-tailed *P*-value of < 0.05 was considered statistically significant.

## Supplementary Information

Supplementary Information.

## Data Availability

The dataset used in this study is under the control of the Data Management Committee of Kyushu University Q-Cohort and cannot be shared publicly due to it containing patient data. However, the dataset is available to researchers who need to use the data for individual patient-level meta-analysis or validation study with another independent cohort. The amended protocol will need to be approved by Kyushu University ethical committee. Requests can be sent to Toshiaki Nakano, M.D., Ph.D., Kyushu University Hospital (toshink@med.kyushu-u.ac.jp).
