# Supplementary material for: Sex differences in the 10-year survival of patients undergoing maintenance hemodialysis in the Q-Cohort Study
Source: Sci Rep. 2022 Jan 10;12:345. doi: 10.1038/s41598-021-03551-x (PMC8748968; doi:10.1038/s41598-021-03551-x)
Supplement: Supplementary file 1 — Supplementary Information. [file 41598_2021_3551_MOESM1_ESM.docx]

**SUPPLEMENTARY MATERIALS ONLINE**

**Sex differences in the 10-year survival of patients undergoing maintenance hemodialysis in the Q-Cohort Study**

Hiroaki Tsujikawa^1^, Shunsuke Yamada^1^, Hiroto Hiyamuta^1^, Masatomo Taniguchi^2^, Kazuhiko Tsuruya^3^, Kumiko Torisu^1,4^, Toshiaki Nakano^1^*, and Takanari Kitazono^1^

Affiliations

^1^ Department of Medicine and Clinical Science, Graduate School of Medical Sciences, Kyushu University, Fukuoka, Japan

^2^ Fukuoka Renal Clinic, Fukuoka, Japan

^3^ Department of Nephrology, Nara Medical University, Nara, Japan

^4^ Department of Integrated Therapy for Chronic Kidney Disease, Graduate School of Medical Sciences, Kyushu University, Fukuoka, Japan

**Correspondence**

Toshiaki Nakano, M.D., Ph.D.

Department of Medicine and Clinical Science, Graduate School of Medical Sciences, Kyushu University, 3–1–1 Maidashi, Higashi-Ku, Fukuoka 8128582, Japan
Tel: +81–92–642–5843

Fax: +81–92–642–5846

E-mail: nakano.toshiaki.455@m.kyushu-u.ac.jp

**SUPPLEMENTARY FIGURES**

**
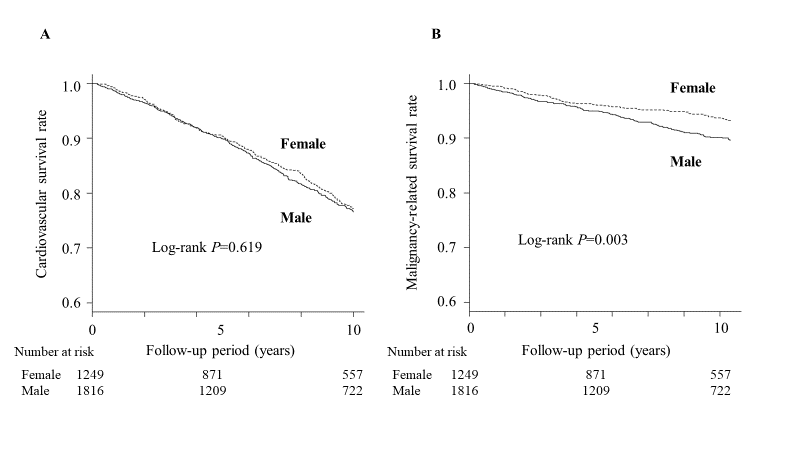
**

**Supplementary Fig 1.** Kaplan-Meier curves for cardiovascular (A) and malignancy-related (B) mortality stratified by sex. A log-rank test was used to determine whether there was a significant difference between women and men. A P-value of less than 0.05 was considered statistically significant.

Table S1. Baseline characteristics after adjusting with the PS-matching method for all-cause death according to sex.

| Variables | males (n = 661) | females (n = 661) | *P-value* | SD |
| --- | --- | --- | --- | --- |
| Age (years) | 64.9 (56.2–72.9) | 64.3 (55.9–73.9) | 0.731 | 0.029 |
| Diabetic nephropathy, n (%) | 187 (28.3) | 190 (28.7) | 0.903 | 0.01 |
| History of CVD, n (%) | 204 (30.9) | 200 (30.3) | 0.858 | 0.013 |
| Dialysis vintage (years) | 5.7 (2.6–11.0) | 5.0 (1.8–11.3) | 0.168 | 0.006 |
| Dialysis time (hours) | 5.0 (4.5–5.0) | 4.5 (4.0–5.0) | <0.001 | 0.451 |
| Body weight (kg) | 54.9 (49.5–61.1) | 48.5 (43.0–54.9) | <0.001 | 0.653 |
| Systolic blood pressure (mmHg) | 153 (140–168) | 152 (138–170) | 0.86 | 0.006 |
| Cardiothoracic ratio (%) | 50.8 (47.6–54.4) | 50.7 (47.0–54.0) | 0.391 | 0.044 |
| nPCR (g/kg/day) | 0.95 (0.85–1.07) | 0.95 (0.84–1.07) | 0.546 | 0.038 |
| Single–pool Kt/V for urea for urea | 1.58 (1.48–1.71) | 1.57 (1.46–1.74) | 0.997 | 0.027 |
| Blood hemoglobin (g/dL) | 10.5 (9.8–11.2) | 10.5 (9.8–11.1) | 0.522 | 0.026 |
| Serum urea nitrogen (mg/dL) | 67 (56–76) | 66 (57–77) | 0.686 | 0.037 |
| Serum creatinine (mg/dL) | 10.7 (9.0–12.4) | 9.3 (7.9–10.9) | <0.001 | 0.619 |
| Serum total cholesterol (mg/dL) | 150 (131–176) | 160 (140–187) | <0.001 | 0.026 |
| Serum albumin (g/dL) | 3.8 (3.5–4.1) | 3.8 (3.6–4.0) | 0.962 | 0.006 |
| Serum CRP (mg/dL) | 0.15 (0.09–0.40) | 0.13 (0.04–0.25) | <0.001 | 0.021 |
| Albumin-corrected serum Ca (mg/dL) | 9.4 (8.9–10.0) | 9.5 (9.0–9.9) | 0.877 | 0.011 |
| Serum phosphate (mg/dL) | 4.8 (4.1–5.6) | 4.9 (4.3–5.7) | 0.262 | 0.044 |
| Serum alkaline phosphatase (U/L) | 238 (183–320) | 238 (185–330) | 0.998 | 0.019 |
| Serum PTH (pg/mL) | 109 (50–221) | 103 (48–219) | 0.608 | 0.052 |
| Use of antihypertensive agents, n (%) | 419 (63.4) | 418 (63.2) | 1 | 0.003 |
| Dose of ESAs, unit/week | 3000 (1500–6000) | 3000 (2000–4500) | 0.389 | 0.001 |
| Use of phosphate binders, n (%) | 551 (83.4) | 554 (83.8) | 0.882 | 0.012 |
| Use of VDRAs, n (%) | 450 (68.1) | 472 (71.4) | 0.209 | 0.073 |

Values are presented as median (interquartile range) for continuous variables and number (percentage) for categorical variables.

The PS was calculated with the following covariates regarding all-cause mortality: age, presence of diabetic nephropathy, history of CVD, dialysis vintage, systolic blood pressure, body weight, cardiothoracic ratio, nPCR, single-pool Kt/V for urea, blood hemoglobin, serum concentration of urea, total cholesterol, albumin, CRP, albumin-corrected serum Ca, phosphate, alkaline phosphatase, and PTH, dose of ESAs, use of antihypertensive agents, phosphate binders, and VDRAs.

Abbreviations: Ca, calcium; CRP, C-reactive protein; CVD, cardiovascular disease; ESAs, erythropoiesis-stimulating agents; nPCR, normalized protein catabolic rate; PS, propensity score; PTH, parathyroid hormone; SD, standardized difference; VDRAs, vitamin D receptor activators.

Table S2. Baseline characteristics after adjusting with the PS-matching method for infection-related death according to sex.

| Variables | males (n = 708) | females (n = 708) | *P-value* | SD |
| --- | --- | --- | --- | --- |
| Age (years) | 64.8 (56.5–73.0) | 64.7 (56.3–73.7) | 0.655 | 0.019 |
| Diabetic nephropathy, n (%) | 200 (28.2) | 207 (29.2) | 0.725 | 0.022 |
| History of CVD, n (%) | 213 (30.1) | 216 (30.5) | 0.908 | 0.009 |
| Dialysis vintage (years) | 5.8 (2.4–12.0) | 5.1 (1.9–11.6) | 0.263 | 0.027 |
| Dialysis time (hours) | 5.0 (4.5–5.0) | 4.5 (4.0–5.0) | <0.001 | 0.426 |
| Body weight (kg) | 55.0 (49.5–61.2) | 48.5 (42.9–55.0) | <0.001 | 0.657 |
| Systolic blood pressure (mmHg) | 153 (140–166) | 154 (137–170) | 0.538 | 0.046 |
| nPCR (g/kg/day) | 0.95 (0.85–1.07) | 0.95 (0.85–1.06) | 0.228 | 0.002 |
| Single–pool Kt/V for urea for urea | 1.57 (1.49–1.71) | 1.56 (1.47–1.74) | 0.845 | 0.039 |
| Blood hemoglobin (g/dL) | 10.5 (9.8–11.2) | 10.4 (9.8–11.1) | 0.493 | 0.016 |
| Serum urea nitrogen (mg/dL) | 66 (56–76) | 67 (56–77) | 0.795 | 0.017 |
| Serum creatinine (mg/dL) | 10.7 (9.0–12.5) | 9.3 (7.8–10.8) | <0.001 | 0.641 |
| Serum total cholesterol (mg/dL) | 151 (131–176) | 160 (140–185) | <0.001 | 0.023 |
| Serum albumin (g/dL) | 3.8 (3.5–4.0) | 3.8 (3.6–4.1) | 0.196 | 0.071 |
| Serum CRP (mg/dL) | 0.13 (0.08–0.40) | 0.13 (0.05–0.25) | <0.001 | 0.043 |
| Albumin-corrected serum Ca (mg/dL) | 9.4 (9.0–10.0) | 9.5 (9.0–9.9) | 0.823 | 0.029 |
| Serum phosphate (mg/dL) | 4.8 (4.1–5.6) | 4.9 (4.2–5.7) | 0.605 | 0.025 |
| Serum alkaline phosphatase (U/L) | 236 (178–318) | 239 (186–327) | 0.612 | 0.004 |
| Serum PTH (pg/mL) | 105 (48–210) | 103 (49–217) | 0.91 | 0.012 |
| Dose of ESAs, unit/week | 3000 (1500–6000) | 3000 (1500–4500) | 0.455 | 0.016 |
| Use of phosphate binders, n (%) | 580 (81.9) | 588 (83.1) | 0.625 | 0.03 |
| Use of VDRAs, n (%) | 476 (67.2) | 506 (71.5) | 0.095 | 0.092 |

Values are presented as median (interquartile range) for continuous variables and number (percentage) for categorical variables.

The PS was calculated with the following covariates regarding infection-related mortality: age, presence of diabetic nephropathy, history of CVD, dialysis vintage, systolic blood pressure, body weight, nPCR, single-pool Kt/V for urea, blood hemoglobin, serum concentration of urea, total cholesterol, albumin, C-reactive protein, albumin-corrected serum Ca, phosphate, alkaline phosphatase, and PTH, dose of ESAs, and use of phosphate binders and VDRAs.

Abbreviations: Ca, calcium; CRP, C-reactive protein; CVD, cardiovascular disease; ESAs, erythropoiesis-stimulating agents; nPCR, normalized protein catabolic rate; PS, propensity score; PTH, parathyroid hormone; SD, standardized difference; VDRAs, vitamin D receptor activators.
